# RF Energy Harvesting and Wireless Power Transfer for IoT

**DOI:** 10.3390/s24237567

**Published:** 2024-11-27

**Authors:** Onel Luis Alcaraz López, Katsuya Suto

**Affiliations:** 1 Faculty of Information Technology and Electrical Engineering, University of Oulu, 90570 Oulu, Finland; 2 Graduate School of Informatics and Engineering, The University of Electro-Communications, Tokyo 183-8585, Japan; k.suto@uec.ac.jp

The rapid proliferation of the Internet of Things (IoT) has transformed modern living by interconnecting billions of devices across industrial, commercial, and domestic sectors. These pervasive IoT systems offer unprecedented capabilities, such as real-time monitoring, automation, and intelligent decision-making. However, the widespread deployment of IoT devices faces a critical challenge: sustaining device operations through sustainable energy provision and management. Traditional battery-powered solutions, with their finite lifespans and the logistical burden of periodic replacement, often prove impractical, especially in dense or remote networks. In response, radio frequency (RF) energy harvesting (EH) and wireless power transfer (WPT) technologies have emerged as pivotal innovations, enabling wirelessly powered systems that extend devices’ lifetimes and reduce their maintenance costs. These technologies may provide scalable solutions for powering sensors, actuators, and edge nodes in numerous scenarios, fostering a more autonomous, resilient, and sustainable IoT infrastructure.

Research in RF-EH and RF-WPT, hereinafter referred to as EH and WPT for simplicity, has evolved significantly, driven by advances in antenna/circuit design and the development of protocols to optimize energy transfer across dynamic conditions. Still, challenges persist in the efficient, scalable, and ecologically sensitive deployment of these systems. This Special Issue, comprising 12 research papers from authors across the globe, addresses these challenges through diverse perspectives, exploring innovations and practical applications while critically examining their trade-offs and environmental impacts. The papers are grouped under five key topics that encapsulate the far-ranging approaches and novel contributions presented in this Special Issue. Together, these works aim to advance our understanding and unlock the transformative potential of RF-based energy solutions within the evolving IoT landscape, contributing to the development of sustainable and resilient wireless ecosystems.

## 1. Advanced Antenna and Rectenna Designs

Innovative antenna and RF circuit designs are crucial for maximizing EH efficiency and is the scope of the work carried out in [1,2]. In [1], Aboualalaa et al. contribute by developing a pattern-reconfigurable antenna that is optimized for EH applications. The antenna operates in the 4.17 to 4.5 GHz range and is capable of electronically steering its radiation pattern across 360 degrees using an RF switch matrix, even switching between directional and omnidirectional modes. The proposed solution achieves high performance, flexibility, and integration capability for IoT environments. Meanwhile, in [2], Abdulwali et al. introduce a high-performance circularly polarized rectenna for EH at 2.45 GHz. The design offers compact design, harmonic rejection, high radiation efficiency (80–91%), stable output for resistive loads, 7.2 dBi directivity, and a conversion efficiency ranging from 36% to 70% for low-input power levels (−10 to 0 dBm).

## 2. Statistical Analysis of EH

Assessing the performance statistics of EH in a variety of scenarios and channel conditions, as reported in [3,4,5], is crucial for predicting and later optimizing a system’s performance in real-world conditions. For instance, the energy harvested from an unmodulated carrier is statistically characterized by Galmés in [3] under generalized-K wireless propagation conditions, capturing the path loss, shadowing, and fading effects. The study provides exact closed-form expressions for the mean and variance of energy harvested over time, offering insights for optimizing WPT in large-scale IoT networks. The results emphasize the need for energy devices that can dynamically handle wide input ranges. Meanwhile, in [4], Le-Thanh et al. derive analytical expressions for the outage probability and throughput of a point-to-point wireless communication system in κ-μ shadowed fading channels, wherein the source harvests RF energy from a dedicated energy transmitter. They reveal that EH nonlinearity has a more severe impact on a system’s performance than hardware imperfections and that communication reliability can be substantially improved through careful tuning of parameters such as the time-splitting factor. Finally, in [5], Kozić et al. analyze cognitive radio networks wherein secondary transmitters are powered by a dedicated energy transmitter and coexist with a primary network under strict interference constraints, factoring in statistical channel state information and random mobility. The study derives analytical expressions for the system’s outage probability, outage capacity, and ergodic capacity, considering the mobility modeled using the random waypoint model in a Nakagami-m fading environment. Their key findings highlight the trade-offs between harvested energy, interference limits, and throughput, emphasizing system optimization through parameters such as the interference threshold, fading characteristics, and mobility patterns, all validated using simulations.

## 3. Optimization and Resource Management in RF-Powered Communication Networks

Ensuring effective utilization of limited system resources is essential to enable more reliable and scalable WPT and EH applications in communication networks, and this is the focus in [6,7,8,9]. Specifically, the integration of WPT into vehicular and IoT communication networks in the presence of multi-antenna eavesdroppers is explored by Ganapathy et al. in [6]. They optimize the power allocation and splitting ratios to enhance both security and EH and highlight key trade-offs under different transmission conditions. Meanwhile, Kulkarni and Joshi provide a modeling framework for RF-powered New Radio Unlicensed networks in [7]. They leverage a three-dimensional Markov chain to characterize and analyze key performance parameters, such as throughput, collision, transmission, and outage probabilities for IoT nodes that are wirelessly powered by a base station. Interestingly, it is shown that the node density and network configuration significantly impact the performance metrics, but the energy storage levels have a minimal effect. Notably, Eidaks et al. demonstrate multi-hop indoor WPT in [8], showcasing practical scenarios and efficiency gains. As their experiments indicate, multi-hop networks based on signal amplification can improve the power reception in line-of-sight and non-line-of-sight scenarios, especially by employing sub-GHz frequencies for a greater range. The authors emphasize the potential of such configurations to optimize power distribution without additional infrastructure, reducing the network complexity and improving coverage. Finally, combining semantic–functional communication with EH is proposed by Silva et al. in [9] to enable efficient multiuser event-driven sensor networks. In a nutshell, sensors transmit meaningful events using energy-based signaling without conventional demodulation, allowing for simultaneous data transmission and battery recharging in an efficient and integrated manner. Their simulation results show the approach can balance energy consumption, event detection, and communication efficiency, evincing promise for future WPT applications requiring real-time responsiveness.

## 4. UAV-Based Solutions and Ecological Assessments

Unmanned aerial vehicles (UAVs) offer potential avenues for power delivery to distributed IoT networks, particularly in challenging environments. However, such potential still requires further assessment to ensure sustainable and practical deployment, especially in terms operational constraints, performance trade-offs, and ecological impacts, several of which are addressed in [10,11]. Specifically, the use of UAVs is considered by Cvetković et al. [10] to restore communication and power in an industrial system during emergencies. They showcase the utility of UAV-assisted simultaneous wireless information and power transfer systems for industrial emergency applications, where maintaining power and data flow under challenging conditions is critical. They also assess how the system’s performance depends on the UAV’s positioning, power allocation, and EH efficiency. For this, and also related to the “EH Statistical Analysis” topic, analytical expressions for the system outage probability and throughput are derived considering Nakagami-m and Fisher–Snedecor fading channels. Meanwhile, Van Mulders et al. present a comprehensive study in [11] on using UAVs to service energy to IoT nodes via recharging or battery replacement. They compare energy efficiencies and ecological impacts between UAV-based recharging, battery replacement, and alternative methods such as remote WPT. For this, the energy consumption of a small license-free UAV is formally characterized, expressions for system efficiencies are derived, and efficient designs/deployments are investigated. The authors show that UAV-based servicing is more energy-efficient over greater distances compared with other methods, with battery swapping generally outperforming recharging due to reduced hovering time. The ecological implications, such as toxic materials and e-waste, are thoroughly assessed, offering insights into sustainable device maintenance practices.

## 5. RF Exposure Mitigation

Mitigating RF exposure is critical for the safe deployment of WPT systems, as high levels of electromagnetic fields may pose health risks and interfere with biological processes. Detecting potential humans/animals in the environment can help in this regard, as this allows for real-time monitoring and dynamic deactivating, beam steering, or adjusting the power transfer when needed, thus ensuring compliance with safety regulations while maintaining efficient energy delivery. A step in this direction is reported by Mattos et al. in [12], wherein multiple millimeter-wave (mmWave) radars are explored for detecting humans and small animals in indoor environments. The authors propose and evaluate different data fusion and radar positioning strategies, demonstrating that combining data from multiple radars significantly enhances the detection sensitivity and precision compared with a single radar.
